# Geographical variation in high-impact chronic pain and psychological associations at the regional level: a multilevel analysis of a large-scale internet-based cross-sectional survey

**DOI:** 10.3389/fpubh.2024.1482177

**Published:** 2024-12-12

**Authors:** Kenta Wakaizumi, Chisato Tanaka, Yuta Shinohara, Yihuan Wu, Saki Takaoka, Morihiko Kawate, Hiroyuki Oka, Ko Matsudaira

**Affiliations:** ^1^Department of Anesthesiology, Keio University School of Medicine, Tokyo, Japan; ^2^Interdisciplinary Pain Center, Keio University Hospital, Tokyo, Japan; ^3^Division of Musculoskeletal AI System Development, Faculty of Medicine, The University of Tokyo, Tokyo, Japan; ^4^Department of Pain Medicine, Fukushima Medical University School of Medicine, Fukushima, Japan

**Keywords:** high impact chronic pain, geographical variation, subjective stress, profile of mood states, multilevel analysis

## Abstract

**Background:**

A geographical analysis could be employed to uncover social risk factors and interventions linked to chronic pain. Nonetheless, geographical variation in chronic pain across different regions of Japan have not been well explored. This study aims to investigate geographical variation in high-impact chronic pain (HICP), defined as moderate to severe chronic pain, and examine the associated psychological factors at the prefecture level.

**Methods:**

A cross-sectional Internet-based survey involving 52,353 participants was conducted to assess chronic pain conditions, stress levels, mood states, educational levels, living status, regions, sleep duration, and exercise habits. A geographical analysis evaluated the prevalence of HICP at the prefecture level, and a multilevel analysis explored the risk factors for HICP at both individual and prefecture levels.

**Results:**

The geographical analysis revealed that Fukushima exhibited the highest HICP prevalence (23.2%; *z*-score = 2.11), Oita ranked second (23.0%; *z*-score = 2.00), and Okinawa showed the lowest prevalence (14.9%; *z*-score = −2.45). Geographical maps of Japan indicated that regional-level subjective stress, negative emotions, and short sleep were associated with higher HICP prevalence. In contrast, positive emotions, such as vigor, were associated with lower prevalence. Multilevel analysis revealed a significant improvement in model fit after incorporating psychological factors at the prefecture level (*p* < 0.001) and identified significant associations between high subjective stress and low vigor at the prefecture level with HICP prevalence (*p* < 0.001).

**Conclusion:**

There are regional differences in HICP prevalence, and at the prefecture level, subjective stress and vigor are associated with HICP.

## 1 Introduction

Chronic pain, defined as persistent pain for at least 3 months (1), is a public health issue with a high prevalence rate and poses a large socioeconomic burden (2). Conditions such as lower back pain, neck and shoulder pain, fibromyalgia, headache, and arthritis are particularly common, leading to both physical and psychological impairment, and diminished quality of life (3). Importantly, chronic pain is a multifactorial experience, with increasing evidence supporting the role of psychosocial factors alongside physical contributors in its development, persistence, and severity (4, 5). Lower back pain, neck and shoulder pain, headache, and arthritis are common chronic pain conditions associated with physical and psychological disability and reduced quality of life. Importantly, chronic pain is multifactorial; therefore, a psychosocial approach, in addition to a physical one, is often necessary for effective treatment and management of pain (6). In fact, several studies have demonstrated that social support is associated with reduced pain severity and less pain-related disability in individuals with chronic pain (7, 8). However, guidelines on chronic pain published by the National Institute for Health and Care Excellence in the United Kingdom (9) concluded that there is no evidence to support the use of social interventions in the management of chronic pain. The committee has recommended further research to obtain high-quality evidence on this topic.

Regional variation in pain have not been well studied, although geographical comparisons could help identify social determinants that influence pain levels. Prefecture is the term for an administrative division in Japan, commonly referring to the 47 regional administrative areas that include Tokyo, Hokkaido, Osaka, Kyoto, and the other 43 prefectures. Prefectures are characterized by different climates, lifestyles, economies, and other factors. Using data from 12,883 individuals aged 65 years and older living in 58 municipalities, Yamada et al. showed that there was a 1.89-fold difference in the prevalence of chronic musculoskeletal pain (10). However, while the municipalities included in this study were distributed across Japan, they did not include all prefectures. Moreover, regional variation in the working-age population was still unclear. Zajacova et al. also identified geographical variation in pain using data from an Internet-based survey in the United States and Canada (11). The difference is partly related to the poorer economic conditions of North Americans. Although the total number of respondents in the above study was relatively small and some states and provinces had only a few respondents, which was not sufficient to fully represent the regions, geographical variation may still indicate social determinants associated with chronic pain.

Thus, studies of regional variation in chronic pain are important to identify social risk factors and clues for social interventions. However, homogeneous, large-scale epidemiological data are required to provide a large enough sample size to calculate the average scores in each region. Japan has a landmass that stretches from east to west and from north to south, a sufficient population, and the necessary infrastructure to collect research data is available throughout the country. In addition, the basic level of education of the population is high enough that questionnaire surveys are feasible for the majority of the population. Therefore, it is possible to investigate regional variation in Japan if large-scale data can be collected using the Internet. This study used previously obtained cross-sectional Internet-based data that included a sufficient number of participants from each prefecture in Japan (12). This study aimed to detect regional differences in moderate-to-severe chronic pain and to examine the psychosocial factors associated with such pain at the prefecture level.

## 2 Methods

### 2.1 Ethical concerns

The present study was approved by the Research Ethics Committee of the University of Tokyo (approval number: 2018132NI) and was conducted following the tenets of the Declaration of Helsinki of 1964 and its later amendments, and an ethical guideline for medical and health research involving human subjects issued by the Japanese Ministry of Health, Labour and Welfare.

### 2.2 Study population

A web-based survey was conducted involving the general population of Japan aged 20–64 years in February 2015. Participants were recruited from approximately 1.37 million individuals across Japan, who have voluntarily registered, through an Internet research company, United Inc. (Tokyo, Japan). Out of the eligible participants, 270,000 individuals were selected for each sex, age, and prefecture category using simple random sampling. They were invited to complete a series of questionnaires online, and the distribution of the questionnaire was completed when the number of respondents met the predetermined target for each category of sex, age, and prefecture, which was established in advance according to the population distribution. After providing their consent, 52,353 people voluntarily responded to the survey without any missing values or personally identifiable information. The mean age of the participants was 42.7 years, with a standard deviation (SD) of 12.1 years, and the proportion of women in the sample was 49.9%.

### 2.3 Measures

Participants reported average pain intensity over the previous 4 weeks on the Numerical Rating Scale (NRS), where “0” corresponds to no pain and “10” indicates the worst possible pain, pain duration (<3 or ≥ 3 months), and painful sites (multiple answers allowed out of three major pain sites: lower back, neck, and knees). High-impact chronic pain (HICP) was defined as moderate-to-severe chronic pain with a pain intensity of ≥4/10 on the NRS and a pain duration of ≥3 months. The remaining individuals with pain were categorized as having low-impact pain (LIP), which is not as severe a pain condition as HICP. All individuals completed the 11-scale subjective stress questionnaire (0: no stress, to 10: worst imaginable) over the past 4 weeks and the Profile of Mood States (POMS)—Brief Form, Japanese version (13). The POMS is a 30-item questionnaire assessing the mood of individuals based on the following six mood construct domains: Tension-Anxiety, Depression-Dejection, Anger-Hostility, Fatigue, Confusion, and Vigor. Each item is rated on a five-point scale, and the score for each domain ranges from 0 to 20; higher scores indicate more disturbances, except for the Vigor domain. Individuals who reported an educational level less than a high-school degree were categorized into a low-education group. The following characteristics were also investigated: body mass index (BMI), smoking status (current smoker or non-smoker), marital status (married, never married, divorced, or widowed), living status (alone or with family), living area (47 Japanese prefectures), sleep duration (<5 h; ≥5, <6 h; ≥6 h; <7 h; ≥7 h, <8 h; ≥8 h; <9 h; or ≥ 9 h), and levels of frequency of at least 30-min of exercise habits in the previous year (at least twice per week; once per week; a few times per month; no exercise habits).

### 2.4 Geographical assessment of participant characteristics and measures

Region-based averages were calculated as an age- and sex-adjusted mean value since there was significant regional variation in age and sex across prefectures. The averages were plotted on a Japanese heat map corresponding to 47 prefectures with respect to the prevalence of HICP and pain-related characteristics, including Subjective Stress, Fatigue, Vigor, Prevalence of High-Frequency Exercise, Tension-Anxiety, Depression-Rejection, Anger-Hostility, Confusion, and BMI, in addition to the proportion of individuals with short sleep, current smoking status, and exercise habits. Each region-based value was standardized to a *z*-score, for which values >2 or <−2 were considered significant. Although educational level, marital status, and living status showed regional variation, we did not perform a geographical assessment on them because they were only a part of the regional characteristics across prefectures.

### 2.5 Multilevel analysis using regional psychological factors

A multilevel analysis was performed to identify an association between risk factors at a prefecture level and the prevalence of HICP at an individual level for the specific factors that were significant (absolute *z*-score ≥ 2) in the geographical analysis at a prefecture level. We used mean values for the psychological factors, which were continuous variables, and prevalence rates for categorical variables as independent variables at the prefecture level.

First, we generated a model of HICP using dependent variables on the individual level (model 1). Age, sex, body mass index, low education, marital status, smoking status, and exercise habits were included in the model as confounding factors. Model fit was assessed by a log-likelihood ratio and Akaike information criterion (AIC). Second, we added the risk factors at the prefecture level to model 1 and compared the model fits using a likelihood ratio test. Finally, we generated model 3 by eliminating a few independent variables that were not significant (*p* ≥ 0.05), from model 2.

### 2.6 Statistical software and map visualization

All statistical tests were two-sided. MATLAB 2016a (MathWorks, Inc., Natick, MA) was used for a multilevel analysis and likelihood ratio test. The other statistical analyses and Japanese color map generation were performed using JMP Pro version 13.2 (SAS Institute Inc., Cary, NC).

## 3 Results

Of the 52,353 participants, there were 10,020 (19.1%) with HICP, 30,640 (58.5%) with LIP, and 11,693 (22.3%) without pain. Table 1 shows a higher proportion of people aged older than 40 years, women, low education, current smokers, high BMI (≥25) and short sleep duration (<6 h), corresponding to the severity of pain. Regarding psychological factors, severe pain conditions showed greater subjective stress, Tension-Anxiety, Depression-Rejection, Anger-Hostility, Fatigue, and Confusion. Instead, people with HICP exhibited a lower proportion of exercise habits (more than twice per week) and Vigor.

**Table 1:** Demographic characteristics and behavioral measures.

	No Pain		LIP		HICP		*p*-value
Participants, *n* (%)	11,693	(22.3)	30,640	(58.5)	10,020	(19.1)	
Age (years), *n* (%)							< 0.001
20–29	2,879	(24.6)	5,728	(18.7)	1,212	(12.1)	
30–39	3,187	(27.3)	7,651	(25.0)	2,227	(22.2)	
40–49	2,405	(20.6)	6,875	(22.4)	2,695	(26.9)	
50–59	2083	(17.8)	7,069	(23.1)	2,836	(28.3)	
60–64	1,139	(9.7)	3,317	(10.8)	1,050	(10.5)	
Women, *n* (%)	5,376	(46.0)	15,231	(49.7)	5,555	(55.4)	< 0.001
Low Education, *n* (%)	6,454	(55.7)	17,810	(58.5)	6,396	(64.3)	< 0.001
Living Alone, *n* (%)	2,215	(18.9)	5,585	(18.2)	1817	(18.1)	0.189
Marital Status, *n* (%)							< 0.001
Never married	4,858	(41.6)	11,411	(37.2)	3,352	(33.5)	
Married	6,106	(52.2)	16,983	(55.4)	5,662	(56.5)	
Divorced	605	(5.2)	1889	(6.2)	856	(8.5)	
Widowed	124	(1.1)	357	(1.2)	150	(1.5)	
Health-related characteristics							
Current smoker, *n* (%)	2,695	(23.1)	7,297	(23.8)	2,897	(28.9)	< 0.001
Exercise habits, *n* (%)	2,333	(20.0)	5,835	(19.0)	1768	(17.6)	< 0.001
Body mass index (kg/m^2^), *n* (%)							< 0.001
<18.5	1,596	(13.7)	3,656	(11.9)	1,211	(12.1)	
≥18.5, <25	8,152	(69.7)	21,088	(68.8)	6,444	(64.3)	
≥25	1945	(16.6)	5,896	(19.2)	2,365	(23.6)	
Sleep duration, *n* (%)							< 0.001
<5 h	1,124	(9.6)	3,447	(11.3)	1845	(18.4)	
≥5, <6 h	3,546	(30.3)	10,485	(34.2)	3,655	(36.5)	
≥6, <7 h	3,905	(33.4)	10,265	(33.5)	2,788	(27.8)	
≥7, <8 h	2,198	(18.8)	4,727	(15.4)	1,173	(11.7)	
≥8, <9 h	639	(5.5)	1,219	(4.0)	357	(3.6)	
≥9 h	281	(2.4)	497	(1.6)	202	(2.0)	
Psychological measures							
Subjective Stress, mean (SD)	3.3	(2.5)	4.3	(2.1)	5.9	(2.1)	< 0.001
Profile of mood States, mean (SD)							
Tension-anxiety	3.0	(4.0)	4.5	(4.2)	6.9	(5.0)	< 0.001
Depression-dejection	2.3	(3.7)	3.4	(4.1)	5.6	(5.0)	< 0.001
Anger-hostility	2.5	(3.6)	3.8	(3.9)	5.8	(4.8)	< 0.001
Fatigue	3.3	(4.2)	5.0	(4.5)	8.2	(5.5)	< 0.001
Confusion	4.8	(2.8)	5.7	(3.1)	7.4	(3.9)	< 0.001
Vigor	4.7	(4.7)	4.9	(4.1)	4.3	(3.7)	< 0.001

High-impact chronic pain (HICP) was defined as pain intensity ≥4/10 and pain duration ≥3 months. The other people with pain were categorized into a group of low-impact pain (LIP). Chi-square test and Kruskal-Wallis test were performed for demographic characteristics, which were categorical data, and psychological measures, which were continuous data, respectively. SD = standard deviation.

**Figure 1:**
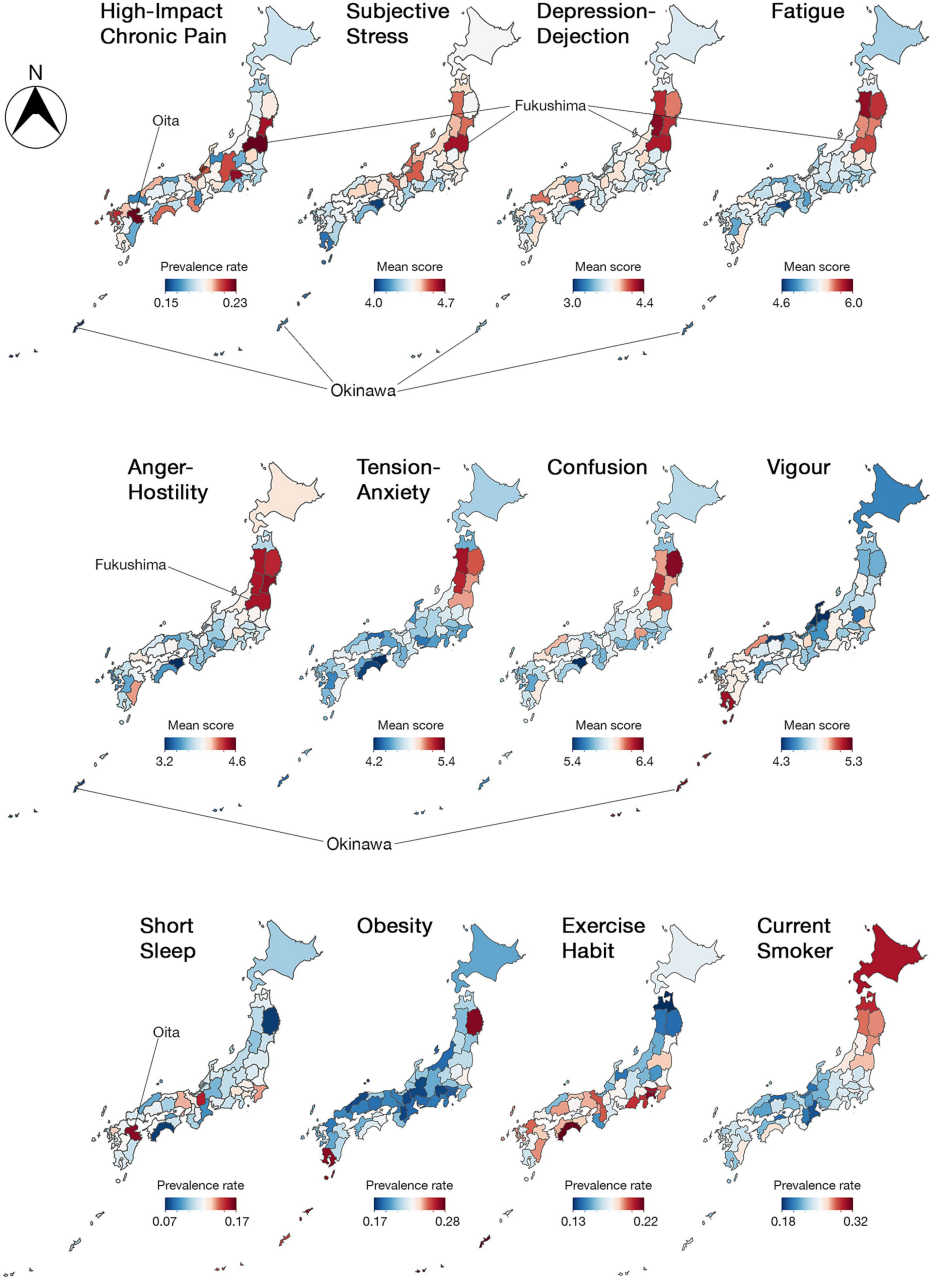
Japanese heat maps representing regional variation in high-impact chronic pain (HICP) and pain-related factors among 47 prefectures. Values are adjusted for age and sex. N, north.

**Figure 2:**
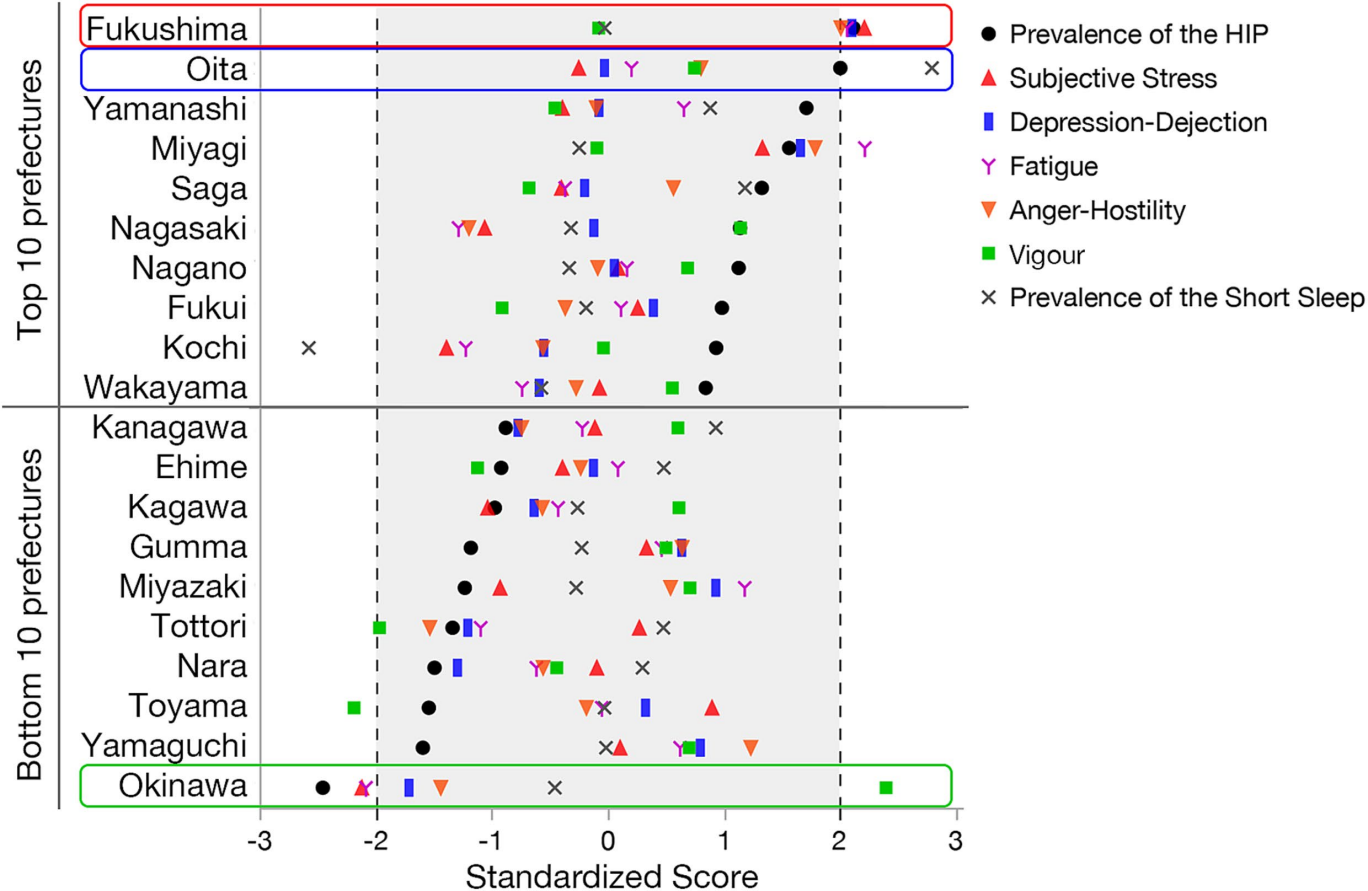
Standardized prevalence rate of high-impact chronic pain (HICP) and short sleep, and scores of subjective stress, depression-dejection, fatigue, anger-hostility, and vigor of each prefecture in Japan. Prefectures with the top and bottom 10 prevalence rates of HICP are presented, with the order sorted by the rate. The *z*-scores in this figure were computed to compare how much the prevalence differed from the average in each region. A score of 0 represents the average, positive scores represent higher-than-average prevalence, and negative ones indicate lower-than-average prevalence. Values greater than 2 or less than −2 were considered significant, and we shaded the *z*–score area from −2 to 2 as a non-significant value.

The geographical analysis revealed that the highest prevalence of HICP (23.2%; *z*-score = 2.11) was recorded in the Fukushima Prefecture with the highest levels of subjective stress and significantly high levels of Depression-Dejection, Fatigue, and Anger-Hostility (6.05, 4.14, 5.85, and 4.44, respectively; *z*-score = 2.21, 2.10, 2.06, and 2.01, respectively; Figures 1, 2 and Supplementary Table S1). Oita Prefecture showed the second highest prevalence of HICP (23.0%; *z*-score = 2.00) with the highest prevalence of people with short sleep duration (16.4%; *z*-score = 2.79). In contrast, Okinawa, the southernmost prefecture in Japan, showed the lowest prevalence of HICP at 14.9% with *z*-score = −2.45 compared with the national average (mean ± SD = 19.4% ± 1.8%). Okinawa also recorded the highest Vigor (4.84; *z*-score = 2.24), along with significantly low Stress and Fatigue levels (4.13 and 4.68, respectively; *z*-score = −2.12 and −2.08, respectively).

Multilevel analysis identified risk factors of HICP at both individual and prefecture levels. Model 1 showed significant associations of subjective stress, Depression-Dejection, Fatigue, Vigor, and short sleep with HICP. Model 2, including risk factors at the prefecture level, showed a significant improvement in model fit (*p* < 0.001) compared to model 1, with the significance of subjective stress and Vigor at the prefecture level. Finally, we eliminated a few variables that were not significant (*p* ≥ 0.05) in model 2, and model 3 showed improved Akaike information criterion (AIC), although the model fit did not significantly improve on the likelihood ratio test (Table 2).

**Table 2:** Multilevel analysis of risk factors for high-impact chronic pain (HICP).

	Model 1		Model 2		Model 3	
	β	(SEM)	β	(SEM)	β	(SEM)
Individual level						
Interception	−1.947	(0.081)***	−4.941	(1.185)***	−4.269	(0.431)***
Subjective stress	0.630	(0.017)***	0.267	(0.007)***	0.267	(0.007)***
Depression-dejection	−0.107	(0.019)***	−0.025	(0.004)***	−0.024	(0.004)***
Anger-hostility	0.014	(0.017)	0.003	(0.004)		
Fatigue	0.473	(0.020)***	0.097	(0.004)***	0.098	(0.004)***
Vigor	−0.063	(0.014)***	−0.015	(0.003)***	−0.014	(0.003)***
Short sleep	0.049	(0.011)***	0.149	(0.034)***	0.148	(0.034)***
Prefecture level						
Mean of the subjective stress			0.780	(0.178)***	0.793	(0.164)***
Mean of the depression-dejection			−0.004	(0.124)		
Mean of the anger-hostility			0.062	(0.082)		
Mean of the fatigue			0.303	(0.202)		
Mean of the vigor			−0.351	(0.107)**	−0.251	(0.066)***
Prevalence of the short sleep			−0.182	(0.201)		
Log likelihood ratio	−21,753		−21,737		−21,740	
Akaike Information Criterion	43,543		43,527		43,523	
Likelihood ratio test			32.08***	(vs. Model 1)	5.83	(vs. Model 2)

Adjustment variables: age, sex, body mass index, low education, marital status, smoking status, and exercise habits. SEM, standard error of the mean. **p < 0.01, ***p < 0.001.

## 4 Discussion

This study revealed that there is a regional difference in the prevalence rate of HICP, and the individual prevalence of HICP was associated with a sociopsychological condition at the prefecture level. The prevalence in the Fukushima prefecture was 1.6 times higher than that in Okinawa, and the largest difference in HICP prevalence was 8.3%. The geographical prevalence using Japanese maps suggests that subjective stress, negative emotion, and short sleep at the regional level may contribute to the prevalence of HICP, whereas positive emotion, such as vigor, may be associated with a low prevalence rate of HICP. However, the multilevel analysis identified only high subjective stress and low vigor at the prefecture level as risk factors for HICP.

Subjective stress is a measure representative of negative emotions and is known to be associated with other negative emotions such as depression, anxiety, anger, fatigue, and short sleep (14–18). Due to the presence of multicollinearity, we considered that only subjective stress was relevant in this study and that other negative emotions at the prefecture level were not detected as a factor of HICP. Therefore, subjective stress may be an indicator for policy measures addressing chronic pain at the regional level.

Fukushima showed the highest prevalence rate of HICP with the highest subjective stress. We can still remember the largest natural disaster, the Great East Japan Earthquake and tsunami, which occurred in Fukushima and the subsequent nuclear accident in 2011. Although the Japanese people have historically experienced various types of natural disasters, such as earthquakes, typhoons, and floods, and have faced many difficulties, the Fukushima disaster has finally overwhelmed them in terms of overall impact. According to population-based mental health surveys involving approximately 210,000 people who had previously lived in the evacuation area in Fukushima (19), the prevalence of psychological distress among adult evacuees, based on the six-item Kessler scale, was quite high compared to the general population of Japan (20). Thus, chronic psychological problems induced by the disaster possibly increased stress levels in Fukushima, resulting in the highest prevalence of HICP as of this survey performed in 2015.

On the other hand, our finding that Okinawa had the lowest prevalence rate of chronic pain was consistent with an Internet-based survey previously performed in Japan (21). Okinawa is composed of geographically isolated islands with a subtropical climate distinct from other Japanese prefectures and has developed its own unique history and culture. These distinctions possibly contributed to the developing of healthier lifestyles and personalities in individuals residing on these islands (22). Consequently, the difference in these backgrounds may have influenced the development of HICP and negative emotions in the residents.

Regional climate and sunlight exposure may influence the prevalence of HICP and are linked to seasonal affective disorder (SAD), a subtype of major depressive disorder or bipolar disorder. SAD is characterized by depressive symptoms that generally occur in the fall and winter and improve in the spring and summer. Living at higher latitudes increases SAD risk due to reduced sunlight (23). Additionally, cold and humid climates may worsen joint pain. A lack of sunlight can lead to vitamin D deficiency, which is associated with musculoskeletal pain. Studies show that patients with fibromyalgia and chronic musculoskeletal pain often have low vitamin D levels, and supplementation may help alleviate pain and improve quality of life (24). Cold seasons also restrict outdoor activities, reducing physical activity and potentially exacerbating chronic pain. The Tohoku region, where anger-hostility was high, including Fukushima as shown in Figure 1, is a cold region, and the northwestern region facing the Sea of Japan is known to have short periods of sunshine. On the other hand, vigor was high in Okinawa, a warmer region, and regional-level vigor was associated with HICP in the multi-level analysis. Thus, as climate and sunshine duration are associated with health problems such as mental illness and lifestyle-related diseases (25, 26), they may be more or less associated with the prevalence of HICP.

Regional differences in the prevalence of HICP can be considered as one aspect of health inequalities. As previous social epidemiological studies have shown that social capital is associated with health inequalities, regional differences in social capital may be a factor contributing to regional differences in HICP. Although there are likely to be large differences in social capital depending on whether one lives in an urban or rural area, Yamada et al. concluded from a study using population density that simple differences such as whether one lives in an urban or rural area cannot explain the prevalence of chronic pain (10). On the other hand, referring to previous literature on mental health, social capital, such as family structure and work relationships, is associated with depression and anxiety disorders (27–29), and regional differences in family and work environments may have influenced regional differences in the prevalence of HICP. Since desk work and heavy work may be risk factors for chronic musculoskeletal pain, differences in industrial structure may also be a factor in generating regional differences in HICP.

Income inequality and economic hardship can directly influence health outcomes, and they have been reported to be a target for social interventions to improve health problems (30). Indeed, daily pain intensity was associated with economic hardship and daily financial worry among 250 women with a chronic musculoskeletal condition (31). Therefore, regional economic inequalities may have detrimental effects on the prevalence of HICP. Given that chronic pain is a social issue, the association between social capital and chronic pain is a topic for further research.

The present study has several limitations, although the findings showed that the prevalence of HICP was associated with psychological status at the community level using large-scale epidemiological data. First, since this is a cross-sectional study, it was challenging to identify causal relationships. As previous studies have shown that pain and negative emotions are interconnected in a vicious cycle (32–34), a bidirectional causal relationship between regional stress levels and the prevalence of HICP must also be considered. Future large-scale longitudinal epidemiological studies are required to identify causality. Second, we had no data on economic status at the regional or individual level. As mentioned previously, economic status, inequalities, and social capital, which are associated with both mental health and pain, should be an important issue for policy measures at the regional level. In areas with lower economic resources, access to timely treatment and pain management may be limited, leading to prolonged pain. Additionally, the accessibility of medical resources and the quality of healthcare systems can also impact whether patients receive appropriate treatment. Future studies may include these factors to better understand the role of socioeconomic and healthcare resources in the prevalence of chronic pain. Third, the present study does not include data on individuals aged 65 years and older. As the prevalence of chronic pain is higher in the older adults, it remains possible that regional disparities may be further increased if the population distribution is considered (10). Consistent with the findings of previous individual-level studies, the trend in the relationship between psychological status and prevalence of chronic pain is unlikely to change even in a survey including the older adults. On the other hand, we may identify novel issues of social capital specific to older adults. However, selection bias due to individual accessibility to an epidemiological survey of older adults should be taken into account when interpreting the results. Finally, regional differences in HICP may be influenced by the quality of healthcare, which was not considered in this survey. Complex chronic pain conditions are often difficult to treat in a single department, and there are cases where interdisciplinary cooperation is required. Medical facilities capable of providing advanced pain treatment are unevenly distributed in urban areas across Japan. Thus, there is much room for further research on the issue of regional differences in chronic pain. Further social epidemiological studies are required to identify them in the future.

In conclusion, the average levels of stress and vigor at the prefecture level may be associated factors for moderate or severe chronic pain. It is suggested that social interventions at the prefecture level could improve the prevalence of chronic pain.

## Data Availability

The data analyzed in this study is subject to the following licenses/restrictions: Ethical approval was obtained under the following statement in the research protocol; data are available upon reasonable request and analyzed data in this study are considered to be available under the permission of the corresponding author and data manager. Requests to access these datasets should be directed to Kenta Wakaizumi, kwaka@keio.jp.
