# Supplementary material for: Geographical variation in high-impact chronic pain and psychological associations at the regional level: a multilevel analysis of a large-scale internet-based cross-sectional survey
Source: Front Public Health. 2024 Dec 12;12:1482177. doi: 10.3389/fpubh.2024.1482177 (PMC11669661; doi:10.3389/fpubh.2024.1482177)
Supplement: Supplementary file 1 [file Table_1.docx]

Supplementary materials

**Title**

**Geographical variation in the high impact chronic pain and psychological associations at a regional level: Multilevel analysis in a large-scale internet-based cross-sectional survey.**

**Supplementary table 1. Region-based standardized scores of prevalence rate of high impact pain, the other characteristics, and behavioral measures.** All scores were averaged across peoples living in each prefecture and standardized after adjustment of age and sex. Order of prefectures was sorted by the prevalence value of the high-impact pain.
